# Triglyceride to HDL cholesterol ratio and risk for gestational diabetes and birth of a large-for-gestational-age newborn

**DOI:** 10.22088/cjim.9.4.368

**Published:** 2018

**Authors:** Shahnaz Barat, Azita Ghanbarpour, Zinatossadat Bouzari, Zohre Batebi

**Affiliations:** 1 Infertility and Reproductive Health Research Center, Health Research Institute, Babol University of Medical Sciences, Babol, Iran; 2 Cellular and Molecular Biology Research Center, Health Research Institute, Babol University of Medical Sciences, Babol, Iran; 3 Clinical Research Development Unit of Rouhani Hospital, Babol University of Medical Sciences, Babol, Iran; 4 Student Research Committee, Babol University of Medical Sciences, Babol, Iran

**Keywords:** Gestational Diabetes, Triglyceride, Cholesterol, Triglyceride/HDL Ratio, Risk Factors, Large for Gestational Age

## Abstract

**Background:**

Using oral glucose for glucose challenge test (GCT) and glucose tolerance test (GTT) is problematic, especially in early pregnancy when the pregnant woman is experiencing gastrointestinal complications. This research seeks to investigate the relationship between the ratio of Triglyceride (TG) to high-density lipoprotein cholesterol (HDL-C) and the risk of gestational diabetes and large for gestational age (LGA) fetus for suggesting a more appropriate index for diagnosis of gestational diabetes.

**Methods:**

The present cross-sectional study investigated pregnant women visiting the Perinatal Clinic of Ayatollah Rouhani Hospital in Babol for prenatal care from September 2015-2016. The GCT was performed on these pregnant women at 24-28 weeks as a screening test and their lipid profile, including HDL-C and TG, was simultaneously assessed after eight to 14 hours of fasting.

**Results:**

Significant differences were observed between women with and without gestational diabetes in terms of mean triglyceride, HDL, LDL/HDL ratio, triglyceride/LDL ratio and triglyceride/HDL ratio. The cut-off point of TG/HDL in the GTT was 4.254 with a sensitivity of 79.07% and specificity of 78%.

**Conclusions:**

According to the results obtained, lipid profile can help predict the risk of gestational diabetes, especially TG/HDL ratio that has a high sensitivity to diagnose gestational diabetes, while, lipid indices could not predict birth of a LGA neonate.

Gestational diabetes mellitus (GDM) refers to any degree of glucose intolerance diagnosed or started during pregnancy (1). The prevalence of GDM has increased in recent years throughout the world, as well as Iran (2). As suggested, many factors play a role in the incidence of gestational diabetes, including diagnostic method, mother’s ethnicity, body composition, age of menstruation onset, family history of diabetes, obesity, and history of neonatal death (3, 4). This common metabolic pregnancy disorder is associated with many maternal and fetal complications (preeclampsia, premature rupture of membranes, preterm delivery, increased risk of cesarean section, hydramnios, fetal macrosomia and low birth weight) (5, 6). Furthermore, more than half of women with gestational diabetes develop diabetes within 20 years (7). Therefore, diagnosis of gestational diabetes is very important in reducing maternal and fetal complications.

Glucose challenge test (GCT) is routinely tested for all pregnant women and at-risk patients take oral glucose tolerance test (OGTT) as well for screening of GDM, but these tests are associated with several problems, such as patients’ intolerance after drinking the glucose solution, vomiting, and gastrointestinal complications, research is continued to find a substitute test (8). Along with several biological changes in pregnancy, lipid metabolism and serum lipid profile alters, as well (9), supposed to be not only due to the changes in body fat but also due to other molecular etiologies, including inflammation and insulin resistance (10). 

For example reduced high-density lipoprotein cholesterol (HDL-C) (9) and triglyceride (TG)/HDL-C ratio (11) have been associated with insulin-resistance. As insulin resistance is the principal physiopathology of GDM (3, 12), different lipid ratios have been used to predict the risk of GDM (13). Given the importance of the early diagnosis of GDM and inadequate information about universal or case-specific screening tests for GDM, and considering the changes in lipid metabolism in patients with GDM and the potential pathophysiology of this disease, conducting further studies such as the present one appears necessary.

Although, the primary goal of this lipid alterations in pregnancy is nutritional supply to the fetus, these changes have been associated with several neonatal adverse outcomes, like pre-eclampsia (14). Previous studies have also shown a significant and positive correlation between mother’s fasting TG levels in late pregnancy and neonatal birth weight, irrespective of the mother’s glucose level or weight (15). 

As birth of large for gestational age (LGA) neonates remains a major problem in GDM with a prevalence of about 30% in diabetic women (16), despite the improvements in prenatal care and the early diagnosis and proper treatment of diabetes, investigating an index for prediction of this neonatal complication is of great value. As previously suggested, hypertriglyceridemia (17) and low HDL-C (18) are associated with LGA, therefore, we hypothesized in the present research that TG/HDL-C ratio could be an appropriate index for prediction of LGA. 

Furthermore, as explained above, TG and HDL-C have been separately associated with the risk of GDM, but the association of TG/HDL-C ratio has not been studied, as far as we are concerned. Therefore, the present study aimed to investigate the association of TG/HDL-C ratio with GDM and LGA to help diagnose or predict these conditions and prevent their complications.

## Methods

The present cross-sectional study was conducted on pregnant women visiting the Perinatal Clinic of Ayatollah Rouhani Hospital in Babol, who referred for prenatal care to this center in September 2015-2016. The study was approved by Ethics Committee of Babol University of Medical Sciences. Thus at first, the study objectives were explained to the eligible women and after signing the written informed consent, the checklist of demographic details was completed for the patients. The study inclusion criteria consisted of having a singleton pregnancy, being in the second trimester when first receiving perinatal care, gestational age of ≥ 36 weeks at delivery, mother’s age between 18 and 35 and complete medical records.

 The exclusion criteria were having type I and II diabetes, documented hyperlipidemia before pregnancy, documented hypertension, cardiovascular diseases, and metabolic syndrome before pregnancy and history of severe systemic diseases such as liver cirrhosis, chronic renal failure, severe anemia, autoimmune diseases and untreated endocrine disorders. The sample size was calculated at 84 at the group with diabetes, considering α=0.05 and 1-β=0.8, based on the study by Wang et al. (13) and 240 in the group without diabetes, thus, considering the chance of lost to follow-up, 100 cases and 300 controls were considered as the final sample size. 

GDM was diagnosed in accordance with the latest revision of American Diabetes Association’s guidelines (19), and GCT screening test was tested for all the included pregnant women at 24-28 weeks of gestation and their plasma glucose was measured one hour after the oral intake of 50 grams of glucose, irrespective of the timing of the last meal. If plasma glucose was ≥140 mg/dl one hour later, the patient was suspected of diabetes and GTT was tested two weeks later using 100 grams of oral glucose. The cut-off point used was based on Carpenter variables for diagnosis of GDM; GDM was diagnosed when two or more of the following were positive: fasting blood sugar >95mg/dl, blood sugar >180 mg/dl one hour after the oral intake of 100 grams of glucose, blood sugar >155 mg/dl two hours later and blood sugar >140mg/dl three hours later (20). HbA1C was also measured in women diagnosed with gestational diabetes. The diabetic women were given advice on physical exercise, diabetic diet, and regular blood sugar monitoring, and insulin therapy was initiated in them if they had unfavorable blood sugar levels. 

The subjects’ lipid profile, including HDL-C (first trimester 40-78, second trimester 52-87, third trimester 48-87) and TG (first trimester 40-159, second trimester 75-382, third trimester 131-453, was checked simultaneously after eight to 14 hours of fasting (definition for pregnant women: hypertriglyceridemia means TG ≥ 1.7 mmol/L or 150 mg/dl and low HDL means HDL-C < 1 mmol/L or 80 mg/dl). All the serum parameters were measured at Ayatollah Rouhani Hospital Laboratory in Babol, by Ziestchem Diagnostic Tehran; according to the glucose kit, the kit’s sensitivity was 5 mg/dL, cv<2%, and r=0.966. According to the manufacturer’s instructions, HDL’s kit had a sensitivity of 1 mg/dL, cv<4%, and r=0.995, and that of TG’s kit had a sensitivity of 5 mg/dL, cv<2%, and r=0.993. Participants were followed-up until child birth and neonatal data, including weight and gender, were recorded upon childbirth. An infant with a birth weight in the high 90^th^ percentile was considered as LGA (21). The data completed for all pregnant women with and without GDM included BMI before pregnancy, gestational age and weight at GCT, gestational age and weight at childbirth, weight gain during pregnancy, blood sugar one hour after oral intake of 50 grams of glucose, HbA1C level, lipid profile (TG/HDL-C ratio) and blood sugar one, two and three hours after glucose intake and while fasting.

 The normal distribution of the data was assessed using K-S test. Chi-square test and t-test were used for comparing the quantitative and qualitative variables and logistic regression test was used for multivariate analysis. After adjusting the groups in terms of the mother's age and BMI, the relationship between birth weight and TG/HDL-C ratio and other variables was assessed, and the risk factor for LGA was determined through statistical tests. The data obtained were analyzed in SPSS-22 and CATmaker software was also used to assess the accuracy of the diagnosis by finding the sensitivity, specificity, positive and negative predictive values and positive and negative likelihood ratios. P<0.05 was considered statistically significant. 

## Results

Comparison of the mean values or ratios of the demographic variables between the two groups of pregnant women with and without GDM based on the GTT is shown in table 1. 

**Table 1:** The maternal and neonatal characteristics in the groups with and without gestational diabetes

**Variables**	With GDM* **Mean±SD**	**GDM** **Mean±SD**	**P value**
**Gestational Age (Week)**	1.12±38.55	0.74±37.77	0.001
**Gestational Age (Week) **			
37> 37<	38 212	26 60	0.002
**Age(Years)**	4.87±27.33	30.49±4.00	0.001
**Age(Years) **			
20-29 30-34 >35	184 66 -	42 43 1	0.001
**BMI(Kg/m** ^2^ **)**	25.72± 4.33	28.5±3.73	0.001
**BMI(Kg/m** ^2^ **) **			
<18.5 18.5-24.9 >25	9 117 124	- 17 69	0.001
**Parity**	0.69± 0.64	0.96± 0.77	0.002
**Gravidity**	1.95± 0.90	2.23±1.03	0.017
**Mode of Delivery**			
NVD Cesarean	117 133	35 51	0.327
**Education**			
<Diploma >Diploma	186 64	72 14	0.077
**Job**			
Housewife Employee	235 15	74 12	0.019
**Sex of Neonate**			
Male Female	127 123	46 40	0.778
**Weight of ** **Neonate (Gram)**	3390.8*±*532.3	3542.3±398.4	0.016
**Weight of Neonate (gr) **			
<2500 2500-4000 >4000	5 236 9	- 78 8	0.001

parity, gravidity, neonatal weight, and maternal occupation. The results of t-test, to compare the mean values of TG, cholesterol, LDL, HDL, LDL-C/HDL-C ratio, TG/LDL-C ratio, and TG/HDL-C ratio between the two groups of women with and without GDM based on GTT, showed significant differences between the two groups in TG, HDL, LDL-C/HDL-C ratio, TG/LDL-C ratio, and TG/HDL-C ratio (table 2).

**Table 2:** Differences in serum lipid concentration between the groups with and without gestational diabetes mellitus (GDM)

**Variables**	**Without GDM** **N=87**	**With GDM** **N=250**	**P value**
Triglyceride(TG)(mg/dl)	205.53± 72.51	275.43*±* 69.33	0.001
Cholesterol (mg/dl)	234.41*±* 132.01	228.82*±* 41.10	0.705
Low density lipoprotein (LDL)	122.57*±*43.35	122.82*±*31.47	0.956
High density lipoprotein (HDL)	66.28*±*25.78	53.30*±*14.88	0.001
LDL/HDL	1.98*±*0.70	2.41 *±*0.85	0.001
TG/ LDL	1.85*±*1.10	2.38*±*0.87	0.001
TG/ HDL	3.38*±*1.54	5.37*±*1.56	0.001

The ROC curves for serum lipids level and lipids ratio in the third trimester of pregnancy associated with GTT and LGS were calculated to compare the accuracy of these variables. 

**Fig 1:**
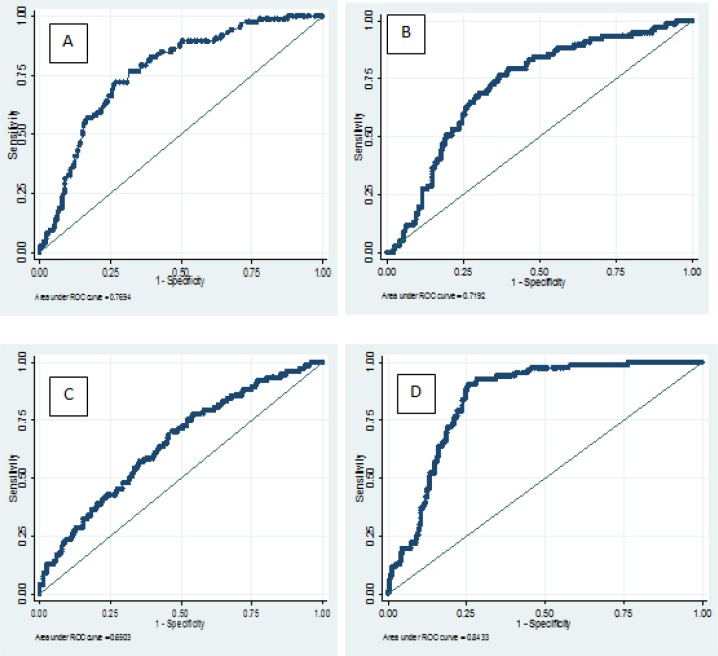
ROC Curve for TG (A), TG/LDL (B), LDL/HDL (C), TG-HDL (D) variables based on GTT test

Table 3 shows the sensitivity, specificity, LR^+^, LR^-^ and odds ratio (OR) estimated for serum lipids level and lipids ratio in GTT and LGA at the optimal identified cutoff value for each method. As shown in table 3 and figure 1A, in the analysis of the ROC curve, the appropriate cut-off point for TG regarding the dependent variable GTT was ~235, at which sensitivity was 72.09% and specificity was 71.6%, and the area under curve (AUC) ROC was 0.769. For LGA, the cutoff point for LDL-C was ~138, at which sensitivity was 53.33% and specificity was 71.38%, and AUC ROC curve was 0.582 (figure 1C).The results of the logistic regression analysis for TG, cholesterol, LDL-C, and HDL-C for GTT and LGA showed that TG had a significant relationship with the dependent variable GDM, based on GTT, with an OR of 1.01 (p<0.001). The regression analysis performed in this study revealed that none of the lipid parameters were helpful in predicting the incidence of LGA (table 4).

**Table 3:** Optimal cutoff points of maternal third-trimester lipids level and lipids ratio for predicting pregnancy outcomes

**Variables**	**Cut off** ***%***	**LR-** ***%***	**LR+** ***%***	**Correctly sorted** ***%***	**Specificity** **%**	**Sensitivity** ***%***	**AUC(95 % CI)**	**Result**
Triglyceride	235 205	0.39 0.98	2.53 1.01	72.92 47.92	71.60 47.65	72.09 52.94	0.769 0.514	GTT Macrosomia
Cholesterol	229 230	0.92 0.66	1.07 1.38	51.84 53.99	51.85 53.40	51.81 64.71	0.516 0.562	GTT Macrosomia
LDL	120 138	1.11 0.65	0.90 1.86	47.2 70.53	49.56 71.38	48.05 53.33	0.480 0.582	GTT Macrosomia
HDL	59 60	1.93 1.15	0.49 0.86	34.23 45.83	34.80 45.77	32.56 47.06	0.266 0.427	GTT Macrosomia

**Table 4:** The relationship between pregnancy complications and maternal serum lipid concentrations in the third trimester

**Variables**	**HDL**		**LDL**		**Cholesterol**		**HDL**		**Triglyceride**	
	**p**	**AOR** **95 % CI**	**p**	**AOR** **95 % CI**	**p**	**AOR** **95 % CI**	**AOR** **95 % CI**	**p**	**p**	**AOR** **95 % CI**
GTT	0.179	0.93( 0.042-1.803)	0.398	1.00 (0.995-1.02)	0.426	1.00 (0.998-1.003)	0.93( 0.042-1.803)	0.179	0.0009	1.01 (1.009-1.018)
Macrosomia	0.378	0.98 ( 0.945-1.021)	0.092	1.01 (0.997-1.029)	0.893	0.99 (0.989-1.008)	0.98 ( 0.945-1.021)	0.378	0.987	1.00(.0992-0.971

## Discussion

The results of this survey on comparison of maternal and neonatal variables between pregnant mothers with and without GDM showed significant differences between the groups in terms of TG, HDL-C, LDL-C/HDL-C ratio, TG/LDL-C ratio, and TG/HDL-C ratio. Similar to the results of our study, various studies have confirmed the association of dyslipidemia in pregnancies complicated with gestational diabetes (22-24). In the present study, the cut-off point (AUC ROC) with the use of GTT for TG was ~235, for LDL-C was ~138, and for TG/HDL-C ratio was 4.254, with a sensitivity of 79.07% and specificity of 78%. 

There are contradictory reports on lipid concentrations and ratios in GDM compared to women with healthy pregnancies. Dos Santos-Weiss et al. reported TG/HDL-C ratio as a predictor of GDM with a sensitivity of 82.6% and specificity of 83.4% at the cut-off point of 0.099 (25), which is consistent to the results of our study, although the values are different that may be attributable to differences in diet, lifestyle and social conditions of the investigated participants. Wang et al. reported that TG/HDL-C ratio, in conjunction with HbA1c and pre-pregnancy BMI, can predict the risk of GDM (13), which is in line with the results of the present study. The second studied outcome in this study included LGA, and regression analysis performed in this study revealed that none of the lipid parameters were helpful in predicting the incidence of LGA, while Wang et al. reported that TG/HDL-C ratio, in conjunction with HbA1c and pre-pregnancy BMI, can predict the risk of LGA (13), and Mosayebi et al. found that maternal FBS and TG levels are independent factors for predicting neonatal weight and LGA (26), which contradict the results of the present study. 

In another study, Sacks et al. showed the association of TG in women with GDM with neonatal weight after adjustment for gestational age (27) and Son et al. found that serum TG concentrations during pregnancy can determine the birth of LGA infants in women with gestational diabetes (17). The inconsistency between the results of the above-mentioned studies with the present study can be attributed to the differences in characteristics of the study population including ethnicity and genetic profile, sample size and method of measuring lipid parameters, which significantly affect neonatal birth weight. Although various studies have confirmed the association of maternal obesity, weight gain during pregnancy and maternal FBS during pregnancy with neonatal LGA (28-30), the role of maternal hyperlipidemia in neonatal development is not yet fully understood. In the present study, lipid parameters were higher in women with GDM. Consistent with the results of the present study, some researchers confirm the significant increase in serum increased lipid profile including concentrations of TG/HDL-C and LDL-C/HDL-C ratios in mothers with GDM compared to healthy pregnancies (31, 32). In the present study, diagnosis of GDM by GTT and GCT showed increased lipid parameters, except for cholesterol and LDL-C. Consistent with the results of our study, Korkmazer et al. found that women with GDM have higher triglyceride levels, while their cholesterol and LDL-C levels are not different from healthy women’s (33), but Lopez-Tinoco et al. found no significant differences between the two groups in terms of serum HDL-C, TC and LDL-C levels, while TG was significantly higher in mothers with GDM (34), and Saucedo et al. showed that TG levels of women with GDM at 24-28 weeks of gestation were significantly higher than women with healthy pregnancies, while no significant differences were observed between the two groups in terms of total cholesterol (35). 

These contradictory results could be attributed to the differences in ethnicity of participants. Moreover, others report no significant differences in serum concentrations of TG, TC, LDL-C, HDL-C and TG/HDL-C ratios between women with and without GDM (36-38). Special attention should be paid to the fact that assessment and interpretation of laboratory parameters during pregnancy is a complicated issue because of the changing levels of various hormones and several metabolic changes, aimed to improve fetal access to nutrition and other factors, which may be impaired or exacerbated in cases with GDM (25). For the same reason, serum tests performed at different trimesters could result in such differences among studies.

**The study limitations: **The limitations of this study include the failure to assess the effect of factors such as maternal obesity and weight gain during pregnancy on the incidence of LGA. Moreover, as lipid profile alters in different weeks of gestation, future studies are recommended to assess the relationship between maternal lipid profile and GDM and LGA at different weeks of gestation.

In conclusion according to the results, TG/HDL-C ratio plays a role in the incidence of GDM and the lipid profile can help predict the risk of GDM, but it cannot help predict the birth of neonate with LGA. The present study also determined the cutoff point for the TG/HDL-C ratio, by GTT, with a sensitivity of 79.07% and specificity of 78%.

## References

[1] Li KE, Cheung Y, Lau B (2014). Use of fasting plasma glucose and haemoglobin A1c in screening for gestational diabetes mellitus in high-risk antenatal patients in Hong Kong. Hong Kong J Gynaecol Obstet Midwifery.

[2] Alfadhli EM, Osman EN, Basri TH (2015). Gestational diabetes among Saudi women: prevalence, risk factors and pregnancy outcomes. Ann Saudi Med.

[3] Schiavone M, Putoto G, Laterza F (2016). Gestational diabetes: An overview with attention for developing countries. Endocr Regul.

[4] Barat S, Bouzari Z, Yazdani S, Moslemi R, Hajian Tilaki K (2016). History of menstrual disorders associated with gestational diabetes mellitus. Caspian J Reprod Med.

[5] Kotanaie M, Bouzari Z, Basirat Z, Kashifard M, Zeinalzadeh M (2012). The comparison of insulin resistance frequency in patients with recurrent early pregnancy loss to normal individuals. BMC Res Notes.

[6] Jensen DM, Korsholm L, Ovesen P (2008). Adverse pregnancy outcome in women with mild glucose intolerance: is there a clinically meaningful threshold value for glucose?. Acta Obstet Gynecol Scand.

[7] Cunningham F, Leveno K, Bloom S (2010). Williams Obstetrics.

[8] Agarwal MM, Punnose J, Dhatt GS (2004). Gestational diabetes: problems associated with the oral glucose tolerance test. Diabetes Res Clin Pract.

[9] Neboh EE, Emeh JK, Aniebue UU (2012). Relationship between lipid and lipoprotein metabolism in trimesters of pregnancy in Nigerian women: Is pregnancy a risk factor?. J Nat Sci Biol Med.

[10] Tinius RA, Cahill AG, Strand EA, Cade WT (2015). Altered maternal lipid metabolism is associated with higher inflammation in obese women during late pregnancy. Integr Obes Diabetes.

[11] McLaughlin T, Abbasi F, Cheal K (2003). Use of metabolic markers to identify overweight individuals who are insulin resistant. Ann Int Med.

[12] Bouzari Z, Elmi F, Esmaeilzadeh S (2016). A Comparison of serum magnesium level in pregnant women with and without gestational diabetes mellitus (GDM). J Babol Univ Med Sci.

[13] Wang D, Xu S, Chen H, Zhong L, Wang Z (2015). The associations between triglyceride to high‐density lipoprotein cholesterol ratios and the risks of gestational diabetes mellitus and large‐for‐gestational‐age infant. Clin Endocrinol.

[14] Spracklen CN, Smith CJ, Saftlas AF, Zhong L, Wang Z (2014). Maternal hyperlipidemia and the risk of preeclampsia: a meta-analysis. Am J Epidemiol.

[15] Schaefer-Graf UM, Graf K, Kulbacka I (2008). Maternal lipids as strong determinants of fetal environment and growth in pregnancies with gestational diabetes mellitus. Diabetes Care.

[16] Lepercq J, Taupin P, Dubois-Laforgue D (2001). Heterogeneity of fetal growth in type 1 diabetic pregnancy. Diabetes Metab.

[17] Son GH, Kwon JY, Kim YH, Park YW (2010). Maternal serum triglycerides as predictive factors for large‐for‐gestational age newborns in women with gestational diabetes mellitus. Acta Obstet Gynecol Scand.

[18] Ornoy A (2011). Prenatal origin of obesity and their complications: Gestational diabetes, maternal overweight and the paradoxical effects of fetal growth restriction and macrosomia. Reprod Toxicol.

[19] Carpenter MW, Coustan DR (1982). Criteria for screening tests for gestational diabetes. Am J Obstet Gynecol.

[20] Toescu V, Nuttall S, Martin U (2004). Changes in plasma lipids and markers of oxidative stress in normal pregnancy and pregnancies complicated by diabetes. Clin Sci.

[21] Gong XM, Li ZH, Yu RJ (2002). Maternal and fetal general parameters: Peoples Medical Publishing House.

[22] Bartha J, Comino-Delgado R, Martinez-Del-Fresno P (2000). Insulin-sensitivity index and carbohydrate and lipid metabolism in gestational diabetes. J Reprod Med.

[23] Clark CM Jr, Qiu C, Amerman B (1997). Gestational diabetes: should it be added to the syndrome of insulin resistance?. Diabetes Care.

[24] Couch SC, Philipson EH, Bendel RB, Wijendran V, Lammi-Keefe CJ (1998). Maternal and cord plasma lipid and lipoprotein concentrations in women with and without gestational diabetes mellitus Predictors of birth weight?. J Reprod Med.

[25] dos Santos-Weiss IC, Réa RR, Fadel-Picheth CM (2013). The plasma logarithm of the triglyceride/HDL-cholesterol ratio is a predictor of low risk gestational diabetes in early pregnancy. Clinica Chimica Acta.

[26] Mossayebi E, Arab Z, Rahmaniyan M, Almassinokiani F, Kabir A (2014). Prediction of neonates' macrosomia with maternal lipid profile of healthy mothers. Pediatrics & Neonatology.

[27] Sacks DA (2007). Etiology, detection, and management of fetal macrosomia in pregnancies complicated by diabetes mellitus. Clin Obstet Gynecol.

[28] Ferber A (2000). Maternal complications of fetal macrosomia. Clin Obstet Gynecol.

[29] Kamanu C, Onwere S, Chigbu B (2009). Fetal macrosomia in African women: a study of 249 cases. Arch Gynecol Obstet.

[30] Ben-Haroush A, Yogev Y, Hod M (2004). Fetal weight estimation in diabetic pregnancies and suspected fetal macrosomia. J Perinatal Med.

[31] Khosrobigi A (2016). Serum values of atherogenic index of plasma and lipid ratios in gestational diabetes mellitus. J Obstet Gynecol Infertil.

[32] Liang Z, Wu Y, Zhu X, Fang Q, Chen D (2016). Insulin resistance and lipid profile during an oral glucose tolerance test in women with and without gestational diabetes mellitus. J Obstet Gynecol.

[33] Korkmazer E, Solak N (2015). Correlation between inflammatory markers and insulin resistance in pregnancy. J Obstet Gynecol.

[34] Lopez-Tinoco C, Roca M, Fernandez-Deudero A (2012). Cytokine profile, metabolic syndrome and cardiovascular disease risk in women with late-onset gestational diabetes mellitus. Cytokine.

[35] Saucedo R, Zarate A, Basurto L (2011). Relationship between circulating adipokines and insulin resistance during pregnancy and postpartum in women with gestational diabetes. Arch Med Res.

[36] Takhshid MA, Haem Z, Aboualizadeh F (2015). The association of circulating adiponectin and+ 45 T/G polymorphism of adiponectin gene with gestational diabetes mellitus in Iranian population. J Diabetes Metabol Disord.

[37] Stein S, Stepan H, Kratzsch J (2010). Serum fibroblast growth factor 21 levels in gestational diabetes mellitus in relation to insulin resistance and dyslipidemia. Metabolism.

[38] Iimura Y, Matsuura M, Yao Z (2015). Lack of predictive power of plasma lipids or lipoproteins for gestational diabetes mellitus in Japanese women. J Diabetes Investig.

